# Discrepancies between Retrospective Review of “Real-Time” Electronic Health Record Documentation and Prospective Observer Documentation of In-Hospital Cardiac Arrest Quality Metrics in an Academic Cardiac Intensive Care Unit

**DOI:** 10.3390/jcm12227102

**Published:** 2023-11-15

**Authors:** Nicholas A. Morris, Cody Couperus, Gregory Jasani, Lauren Day, Christa Stultz, Quincy K. Tran

**Affiliations:** 1 Department of Neurology, University of Maryland School of Medicine, Baltimore, MD 21201, USA; 2 Program in Trauma, R Adam Cowley Shock Trauma Center, University of Maryland School of Medicine, Baltimore, MD 21201, USA; qtran@som.umaryland.edu; 3 Division of Neurocritical Care and Emergency Neurology, University of Maryland Medical Center, Baltimore, MD 21201, USA; 4 Department of Emergency Medicine, University of Maryland School of Medicine, Baltimore, MD 21201, USA; ccouperus@som.umaryland.edu (C.C.); gjasani@som.umaryland.edu (G.J.); lday@som.umaryland.edu (L.D.); 5 Department of Cardiovascular Medicine, University of Maryland Medical Center, Baltimore, MD 21201, USA; cstultz@umm.edu

**Keywords:** IHCA, cardiac arrest, documentation, concordance

## Abstract

Background: Every year, approximately 200,000 patients will experience in-hospital cardiac arrest (IHCA) in the United States. Survival has been shown to be greatest with the prompt initiation of CPR and early interventions, leading to the development of time-based quality measures. It is uncertain how documentation practices affect reports of compliance with time-based quality measures in IHCA. Methods: A retrospective review of all cases of IHCA that occurred in the Cardiac Intensive Care Unit (CICU) at an academic quaternary hospital was conducted. For each case, a member of the code team (observer) documented performance measures as part of a prospective cardiac arrest quality improvement database. We compared those data to those abstracted in the retrospective review of “real-time” documentation in a Resuscitation Narrator module within electronic health records (EHRs) to investigate for discrepancies. Results: We identified 52 cases of IHCA, all of which were witnessed events. In total, 47 (90%) cases were reviewed by observers as receiving epinephrine within 5 min, but only 42 (81%) were documented as such in the EHR review (*p* = 0.04), meaning that the interrater agreement for this metric was low (Kappa = 0.27, 95% CI 0.16–0.36). Four (27%) eligible patients were reported as having defibrillation within 2 min by observers, compared to five (33%) reported by the EHR review (*p* = 0.90), and with substantial agreement (Kappa = 0.73, 95% CI 0.66–0.79). There was almost perfect agreement (Kappa = 0.82, 95% CI 0.76–0.88) for the initial rhythm of cardiac arrest (25% shockable rhythm by observers vs. 29% for EHR review, *p* = 0.31). Conclusion: There was a discrepancy between prospective observers’ documentation of meeting quality standards and that of the retrospective review of “real-time” EHR documentation. A further study is required to understand the cause of discrepancy and its consequences.

## 1. Introduction

Patients who sustain sudden cardiac arrest are associated with high morbidity and high mortality [1]. The 2023 update from the American Heart Association reported that out of approximately 146,000 patients who suffered out-of-hospital cardiac arrest (OHCA) in the United States, only 13,000 (9.1%) survived to hospital discharge, and only 7.1% of patients were discharged from the hospital with good functional status. However, functional impairment continues with these patients for up to 12 months after OHCA, as only 72% of the survivors would be able to return to work after 12 months [1]. Reasons for low rates of survival to hospital discharge or survival with good functional status included the delayed recognition of cardiac arrest, prolonged state of no cardiac activities, low rates of chest compression, or cardioversion from bystanders [1].

On the other hand, every year, approximately 200,000 patients will experience cardiac arrest while being hospitalized for the treatment of other conditions, referred to as in-hospital cardiac arrest (IHCA) in the United States [2]. Unfortunately, even though these patients are already in a hospital when cardiac arrest occurred, their survival remains low at approximately 20% [3], and only 12.9% of IHCA survived to hospital discharge with a good functional status [1]. Although only 12.9% of patients suffering IHCA had an initial cardiac rhythm of ventricular fibrillation (VF) or ventricular tachycardia (VT), according to a 2021 Get-With-The-Guidelines (GWTGs) report [1], up to 65% of patients with these shockable rhythm survived to hospital discharge, compared with approximately 20% of patients with initial pulseless electrical activities (PEAs) or asystole (non-shockable rhythm) [4].

Survival depends on the prompt initiation of cardiopulmonary resuscitation (CPR) [5], early defibrillation for shockable rhythms [6], and the timely administration of epinephrine for non-shockable rhythms [7]. After a cardiac arrest is recognized, whether out-of-hospital or in-hospital, prompt chest compression and the initiation of CPR was considered to be the most important action among the Chain of Survival for patients’ survival and good neurological outcomes. From a study involving a large number of patients who were witnessed to have OHCA, each incremental delay of CPR would decrease the neurologically favorable 1-month survival probability by 8% for patients with shockable rhythm, 4% for PEA, and 6% for asystole, respectively [8]. Time from the recognition of cardiac arrest and defibrillation for shockable rhythm was another aspect considered for the chain of survival among patients with any type of cardiac arrest. After 4 min from cardiac arrest to shockable rhythm, each minute of delay in defibrillation was associated with a statistically lower odds of survival to hospital discharge [6]. For patients who suffered IHCA and had non-shockable rhythm, their chain of survival also depended on the early administration of epinephrine. From a post hoc analysis of prospectively collected data from the multi-institution registry GWTG [7], the administration of epinephrine after the first 3 min when an IHCA event occurred was associated with a significantly lower likelihood of survival to hospital discharge.

Based on this evidence, the current Advanced Cardiovascular Life Support (ACLS) guidelines recommend defibrillation within 2 min after event recognition for shockable rhythms and epinephrine administration within 5 min of event recognition for non-shockable rhythms [5]. Accordingly, the American Heart Association has released quality metrics to improve adherence to evidence-based cares and ultimately save lives [9].

While resuscitation quality metrics are well defined, best documentation practices are not. Studies of resuscitation documentation reveal frequent missing or discrepant data [10]. This study reported that up to 50% of patients did not have a freestanding IHCA clinical document, and those patients were less likely overall to have clinical details of the IHCA available. Time-to-event documentation is particularly challenging [11,12], as well as other paper-based documentation, during IHCA events. The unlikely intervals of 0 min from cardiac arrest recognition to any ACLS interventions were documented in 11.5% of patients suffering IHCA based on the data from the National Registry of Cardiopulmonary Resuscitation [11].

Technologies such as smartphone- or tablet-based applications [13] and electronic health record (EHR) modules [14] have been developed to facilitate real-time documentation to improve code documentation accuracy. In a study involving the simulation of IHCA, when compared to the gold standard of video records, tablet-based documentation had 88% sensitivity, while paper-based documentation only had 68% [13]. Although documentation using EHRs seems promising, few authors have suggested that these solutions had the desired effect in improving code documentation accuracy, as most nurse participants still did not document the IHCA in real-time [14]. Thus, it remains uncertain whether the “real-time” documentation of IHCA events into the EHR at the time of IHCA events would accurately report IHCA quality metrics.

To address this question, we compared the reporting of key IHCA quality metrics by an observer of the event in a prospective, internal quality improvement database to retrospectively review EHRs from a “real-time” code narrator module.

## 2. Methods

### 2.1. Study Settings and Participants

We performed a retrospective review of IHCA events that occurred in the Cardiac Intensive Care Unit (CICU) at a quaternary academic medical center from 1 June 2020 to 30 June 2023. The survival rates were 42% and 24% for IHCA with shockable rhythm or PEA/asystole at the CICU during the study period, respectively. We identified events from a prospectively collected, unit-based, cardiac arrest quality improvement database. For this quality improvement database, nursing members of the CICU quality improvement council, when participating in an IHCA event, would identify themselves as present for the event and document the relevant quality metrics in real-time. After the IHCA event, these nursing members of the CICU quality improvement council were encouraged to enter data from the events that they directly observed (and identify themselves as present for the event). Thus, only IHCA events that had documentation from a member of the CICU quality improvement council were eligible for this study. We excluded events that did not have both documentation by someone who directly observed the event and the EHRs about the event. If a patient experienced cardiac arrest more than once and had both instances reviewed by a code observer, then these codes were counted as two separate events.

This study was exempted from formal consent by the Institutional Review Board (HP-00099858).

### 2.2. Data Documentation during Code Events

At our institution, all CICU nurses are certified in Advanced Cardiac Life Support (ACLS) and undergo training in the use of the Resuscitation Narrator, a real-time module for documenting code events available in the EHRs (Epic Systems, Madison, WI, USA). At the beginning of each clinical shift, each working nurse would be assigned a role during an event if an IHCA event occurred. Nursing members of the Quality Improvement Council were also assigned active clinical roles during the IHCA event, but they would also identify themselves as an observer for the quality improvement purpose. According to these pre-assigned roles during each IHCA event, one CICU nurse would be identified as responsible for documenting directly in the Resuscitation Narrator in real-time when any interventions occurred. The documenting nurse recorded the time of arrest, initial cardiac arrest rhythm, time of defibrillation, time of epinephrine administration, intubation during the code event, the utilization of end-tidal carbon dioxide (ETCO_2_) monitoring, and time of Return of Spontaneous Circulation (ROSC) (or death) in the Resuscitation Narrator.

### 2.3. Prospective Quality Improvement Documentation of Code Events

The observing nurses who documented a code event in the prospective quality improvement database were not blind to the documentation in the Resuscitation Narrator. In this quality improvement database, quality measures such as time to defibrillation < 2 min for shockable rhythms from the recognition of cardiac arrest for shockable rhythms and time to epinephrine < 5 min for non-shockable rhythms were captured as dichotomous (yes/no) variables without the documentation of exact timing. The involvement of code observers in the review process enabled the rectification of apparent inaccuracies in the Resuscitation Narrator-generated documentation. For instance, in cases where the nurse involved in the code was confident that epinephrine had been administered within the first five minutes, even if not explicitly documented as such, the code record was amended to accurately reflect this detail.

### 2.4. Retrospective Review of EHRs

Two study authors (CC, LD) subsequently conducted a review of the Resuscitation Narrator-generated data in the EHRs. Time-to-event data were generated by subtracting the time of arrest from the time of event (i.e., time of defibrillation).

### 2.5. Outcome Measures

The primary outcome of this pilot study was the rate of discrepancy between the critical quality variables, as documented by the directly observed prospective quality improvement database, and the documentation in the “real-time” Resuscitation Narrator-generated EHRs. Variables of interest included initial arrest rhythm, arrest duration, time to defibrillation < 2 min (for shockable rhythms), time to epinephrine administration < 5 min (non-shockable rhythms), intubation during code, and utilization of ETCO_2_ monitoring.

### 2.6. Statistical Analysis

No sample size calculation was performed for this pilot study. The dataset was analyzed using the Python programming language. We provided descriptive analyses to describe our patient population. For the descriptive data, we reported continuous variables as the mean and standard deviation. Categorical variables were reported as the number and proportion for each category in the variable. A two-sided t-test was used to compare continuous variables, while the Chi-squared test was used for categorical data. To further assess the agreement of the prospective data, as collected by the observers, and the documented data, we used Cohen’s Kappa score with bootstrapping using the percentile method to calculate the confidence intervals for categorical variables. A Cohen’s Kappa score of <0.2 would indicate slight agreement between the dataset, a Kappa score 0.21–0.4 indicates fair agreement, 0.41–0.6 moderate agreement, and 0.61–0.8 substantial agreement. Finally, a Cohen’s Kappa score of 0.81–1.0 would indicate almost perfect agreement. All comparisons with a *p*-value < 0.05 were considered statistically significant.

## 3. Results

In total, there were 146 cases of IHCA in the CICU during the study period. Of these, 52 patients’ records were documented in the prospective quality improvement database by a code observer and were included in the study for analysis. The mean (±SD) age for the patients was 67 (±10 years) (Table 1). All arrests were witnessed, and 43/52 (83%) were determined to have cardiac etiology (Table 1).

The prospective database reported 47/52 (90%) patients receiving epinephrine within 5 min of cardiac arrest with non-shockable rhythm compared to 42/52 (81%) patients reported in the EHRs (*p* = 0.02). The Kappa score was 0.27 (95% CI 0.16–0.36), which demonstrates that the interrater agreement between the observers and the documented data, for the time of the epinephrine administration, was only fair (Table 2). Similarly, the Kappa for the interrater agreement for the number of patients who had shockable rhythm and received defibrillation within 2 min from cardiac arrest was 0.73 (95% 0.66–0.79).

The prospective quality improvement database reported 13 (25%) patients who had cardiac arrest with the initial rhythm of ventricular fibrillation or ventricular tachycardia. The electronic health records stated 15 (29%) patients (*p* = 0.31) with these initial shockable rhythms. The Kappa score was 0.82 (95% CI 0.76–0.88). This Kappa score suggested substantial agreement between the code observers and the real-time EHRs.

The average (±SD) for the code duration documented by the prospective database was 17 min 58 s (±12 min, 02 s) and that by EHRs abstraction was 18 min 50 s (±21 min, 22 s). This difference was not statistically significant (*p* = 0.84) (Table 2).

There was also statistically significant difference about the report of the rates of use of ETCO_2_ during the code (*p* = 0.001) (Table 2). The interrater agreement for this use was Kappa 0.04 (95% CI −0.02 to 0.11). However, the interrater agreement for the rate of intubation was good as the Kappa score was 0.68 (95% CI 0.62–0.76) (Table 2).

## 4. Discussion

We found a statistically significant difference in the proportion of patients with non-shockable rhythms reported as meeting the American Heart Association quality measure of receiving epinephrine within five minutes of event recognition. Data from a prospectively collected quality improvement database with data entered by a direct observer suggested a higher proportion of patients meeting this benchmark compared to documentation from a retrospective review of a “real-time”-generated EHR module. We did not find significant differences in other important quality metrics, such as the proportion of patients with shockable rhythms that were defibrillated within two minutes of arrest recognition; however, the reports were not 100% concordant.

The accurate documentation of code data is important as it serves multiple purposes. It facilitates structured debriefing, identifies improvement opportunities, and enables the benchmarking of metrics over time to assess quality improvement initiatives [15]. When code teams attended structured debriefing sessions regularly, quality of care improved. As reported by Hunt et al. [15], structured debriefing sessions that focused on different aspects of codes such as delay of care, pauses in chest compression, and other institutional issues (availability of defibrillator, ETCO_2_ monitors) helped to improve the teams’ performance according to the American Heart Association’s CPR guidelines during pediatric code events. One of the important areas of CPR that benefitted the most from these debriefing sessions was the quality of CPR, such as the rate of compression and depth of compression, both of which were statistically significantly improved after the introduction of weekly structured debriefing sessions.

The regulatory reporting of code data has contributed to registry analyses that inform large-scale shifts of focus to and from various aspects of IHCA care [16]. A large study from the multi-institutional GWTG registry outlined rates of adherence to various aspects of the AHA’s CPR guidelines. Roessler et al. [16] reported that, based on the documentation from the GWTG registry, the rate of adherence to epinephrine administration ≤ 5 min increased from 93% in 2006 to 98% in 2018. However, the rate of adherence of defibrillation ≤ 2 min from event recognition of shockable rhythm only increased from 72% in 2006 to 75% in 2018, despite the fact that patients were already in an ICU at the time of the code event. Thus, the accurate documentation of code events during IHCA from a large registry showed that there are still lots of education opportunities for IHCA care, enabling researchers to assess overall rates of adherence to the AHA’s guidelines. This information will allow for further recommendations to be made to improve guidelines adherence and patients’ outcomes. Additionally, with the development of Artificial Intelligence (AI) and machine learning algorithms, the need for accurate documentation is even more important [17]. The use of AI and machine learning can introduce prediction models and the analysis of large-scale data to provide insights on how to optimize treatment, improving workflow during code events. However, the deployment of AI and machine learning algorithms would require a rigorous validation process, of which accurate documentation is essential to avoid erroneous prediction models, of the guidelines for clinical practice [17].

Finally, accurate reporting can protect providers against malpractice claims and reduce liability risk [18]. The increasing availability of electronic documentation may protect clinicians against mitigation when the standard of care was achieved and there was no medical negligence, especially since courts have allowed the use of clinical practice guidelines as evidence of the standard of care [18]. Therefore, erroneous data documentation in EHRs would increase the risk of liability during code events. Although the number EMR-related claims was small at 1%, compared to most other reasons, the majority (64%) of these EMR-related claims were found to involve user errors [18], stressing the need for the accurate documentations of code events.

Our results build on a previous survey [14] of over 432 inpatient nurses, in which 23% of nurses reported participating in a code inaccurately documented in the EHRs using the same “real-time” code documentation module utilized in our institution. Our study’s results offer quantitative evidence to substantiate those nurses’ assertions. In addition, most of the nurse participants of the survey by Whalen et al. [14] admitted to not using the module as intended, and instead retroactively documenting in the EHR module after the event. Nearly 43% of the nurses believed that retroactive EHR charting increased accuracy over real-time performance. They cited inability to navigate quickly or find the right place to document within the EHR module, delays in computer log-in, and the ability to verify information with other members of the code team as reasons.

In contrast, studies of real-time electronic documentation in simulated environments have found it to be superior to paper-based documentation [13,19]. The use of EHRs, when compared with paper-based documentation for IHCA, was able to capture 24% of the more critical information during simulated code events [19]. Additionally, compared to the gold standard of the video review of code events, entering data in real-time into a tablet during code simulations was associated with a discrepancy of only 3 s of the time from event recognition to all interventions, compared with 77 s (*p* < 0.001) for paper-based documentation. Specifically, the time discrepancy from event recognition to defibrillation from real-time data entering into EHRs was 1 s, compared to 73 s (*p* < 0.001) of paper documentation [13]. The generalizability of these studies is limited due to the simulation paradigm, which may not accurately recreate the unique environmental stressors of an actual, live cardiac arrest event. A smaller study of a tablet-based app compared the use of the app in six real life IHCA events to 12 events documented on paper. Using time-stamped data from the defibrillator as the gold-standard, the study found that data accuracy was significantly higher for the recorded rhythm and utilization of ETCO_2_ monitoring in the electronic documentation group, but not for time to CPR nor code duration. Data completeness was not significantly higher in the electronic documentation group. Unfortunately, no shockable rhythms were included in the study to compare time to defibrillation. Time to epinephrine administration was noted to be a particularly difficult variable to extract using both electronic- and paper-based systems [20]. However, these studies’ findings and our study’s reports suggest that using EHRs for entering code events in real-time would improve accuracy in capturing critical events, and interventions, according to the AHA’s CPR guidelines.

Our study, in which observers had access to the electronic documentation system but evidently concluded that it was mistaken, casts doubt over the absolute superiority of electronic code record keeping. Of course, we cannot be sure of the ground truth. Electronic documentation via defibrillators equipped with accelerometers may offer some assistance, but they cannot accurately determine the time of the arrest onset if they are not already turned on and attached to the patient, nor can defibrillators record data outside their inputs, such as epinephrine administration. Emergency medications like epinephrine are typically included on “override” lists, which allow for administration without processing through a barcode medication administration that timestamps administration into the EHRs. In emergency settings, observed medication administration times differ substantially from the documented times in the EHRs, especially in sicker patients [21]. We posit, therefore, that time-to-epinephrine administration should be interpreted with particular caution. We suggest that novel methods are required to accurately timestamp medications during IHCA events.

This is a retrospective review of data from one ICU at one institution. It is uncertain how generalizable the findings may be. It must be said, however, that we did use the code reporting module on the EHRs with the largest hospital market share. We did not capture the experience of the documenting nurse who utilized the code reporting module. Experience has been shown to increase comfort with the code narrator module, with ICU nurses endorsing more comfort with the narrator module than medical ward nurses [14]. By virtue of working in the CICU, with the ICU in our hospital having the highest frequency of IHCA events, we can expect that our documenting nurses were more experienced than average nurses. In addition, all of our nurses receive formal training in code documentation using the electronic code narrator module that incorporates simulation.

## 5. Conclusions

Our study reported poor interrater agreement between EHR documentation and code observers’ prospective and real-time documentation regarding epinephrine administration and ETCO_2_ usage during IHCA events. However, interrater agreements for the documentation of other IHCA metrics were high, which suggested that the use of EHRs may help to improve the accuracy of IHCA documentation. The disagreement of documentation during IHCA points to some of the challenges that are faced by healthcare professionals in providing accurate documentation for such high-stress and time-sensitive events, but the use of EHRs may help to improve the accuracy of IHCA documentation. While efforts to improve documentation are certainly warranted, there should also be caution when interpreting IHCA quality metrics.

## Figures and Tables

**Table 1:** Demographic information of patients undergoing IHCA and whose charts were documented by both a code team member and an observer at the code event, then reviewed by researchers.

Variables			
Age, years, mean (SD)	67 (10)		
Race, N, %, (95% CI)			
White	26	0.50	0.36–0.64
Black	21	0.40	0.27–0.54
Other	4	0.08	0.00–0.15
Witnessed arrest, N (%), (95% CI)	52	1.00	1.00–1.00
Arrest from cardiac etiology, N (%), (95% CI)	43	0.83	0.72–0.93
Past medical history, N (%), (95% CI)			
Heart failure	46	0.88	0.80–0.97
Respiratory failure	21	0.40	0.27–0.54
Any renal insufficiency	31	0.60	0.46–0.73
Any hepatic insufficiency	14	0.27	0.15–0.39
Any metabolic abnormality	13	0.25	0.13–0.37
Diabetes mellitus	19	0.37	0.23–0.50
Any type of stroke	12	0.23	0.12–0.35
Pneumonia	5	0.10	0.02–0.18
Previous sepsis	6	0.12	0.03–0.20
Any malignancy	3	0.06	0.00–0.12

**Table 2:** Interventions for patients with in-hospital cardiac arrest, as documented by abstraction from the electronic health record or by observers.

Categorical Variables	Abstracted from Electronic Health Record		Documented by Observer		Comparison between Researchers and Observers	
					*p*	Kappa (95% CI)
Epinephrine within 5 min (N, %)						
Yes	42	0.81	47	0.90	0.02	0.27 (0.16–0.36)
No	7	0.13	0	0.00		
Not indicated	3	0.06	5	0.10		
Shock within 2 min, (N, %)						
Yes	5	0.10	4	0.08	0.90	0.73 (0.66–0.79)
No	10	0.19	9	0.17		
Not Shockable	37	0.71	39	0.75		
Initial rhythm of cardiac arrest, (N %)						
PEA/Asystole	35	0.67	39	0.75	0.31	0.82 (0.76–0.88)
Unknown	2	0.04	0	0.00		
VT/VF	15	0.29	13	0.25		
Intubation during code, (N, %)						
Yes	24	0.46	18	0.35	0.32	0.68 (0.62–0.76)
No	28	0.54	34	0.65		
ETCO_2_ during code, (N, %)						
Yes	40	0.77	20	0.38	0.001	0.04 (−0.02 to 0.11)
No	12	0.23	32	0.62		
Duration of codes, mean (SD), (hour/minute/second)	0:18:50 (00:21:22)		0:17:58 (00:21:23)		0.84	NA

95% CI, 95% confidence interval; ETCO_2_, end-tidal carbon dioxide monitoring; NA, not applicable; VT, ventricular tachycardia; VF, ventricular fibrillation; PEA, pulseless electrical arrest.

## Data Availability

Data are contained within the article.
